# Bile duct‐ligated mice exhibit multiple phenotypic similarities to acute decompensation patients despite histological differences

**DOI:** 10.1111/liv.12876

**Published:** 2015-06-22

**Authors:** Alastair O'Brien, Louise China, Karen A. Massey, Anna Nicolaou, Alison Winstanley, Justine Newson, Adrian Hobbs, Tatsiana Audzevich, Derek W. Gilroy

**Affiliations:** ^1^ Centre for Clinical Pharmacology and TherapeuticsDivision of MedicineUniversity College LondonLondonUK; ^2^ Manchester Pharmacy SchoolFaculty of Medical and Human Sciencesthe University of ManchesterManchesterUK; ^3^ Department of HistopathologyUniversity College London HospitalsLondonUK; ^4^ St. Bart's & the London Medical SchoolLondonUK

**Keywords:** bile duct ligation, carbon tetrachloride, eicosanoids, immune suppression, leucocyte trafficking

## Abstract

**Background & Aims:**

Patients with decompensated cirrhosis are susceptible to infection. Innate immune dysfunction and development of organ failure are considered to underlie this. A rodent model of liver disease sharing these phenotypic features would assist *in vivo* study of underlying mechanisms and testing of therapeutics. We evaluated three models to identify which demonstrated the greatest clinical and immunological phenotypic similarity to patients with acutely decompensated (AD) cirrhosis.

**Methods:**

We selected Bile Duct Ligation (BDL) rats at 4 weeks, BDL mice at 14 days and Carbon tetrachloride (CCl_4_) mice at 10 weeks (with studies performed 7 days after final CCl_4_ infection). We examined organ dysfunction, inflammatory response to carrageenan‐in‐paw, plasma eicosanoid concentrations, macrophage cytokine production and responses to peritoneal infection.

**Results:**

Bile duct ligation caused sarcopenia, liver, cardiovascular and renal dysfunction whereas CCl_4_ mice demonstrated no clinical abnormalities. BDL rodents exhibited depressed response to carrageenan‐in‐paw unlike CCl_4_ mice. BDL rats have slightly elevated plasma eicosanoid levels and plasma showed partial PGE_2_‐mediated immune suppression whereas CCl_4_ mice did not. Plasma NOx was elevated in patients with acute or chronic liver failure (AoCLF) compared to healthy volunteers and BDL rodents but not CCl_4_ mice. Elevated nitric oxide (NO) via inducible nitric oxide synthase (iNOS) mediates defective leucocyte trafficking in BDL rodent models.

**Conclusions:**

We conclude that BDL mice and rats are not simply models of cholestatic liver injury but may be used to study mechanisms underlying poor outcome from infection in AD and have identified elevated NO as a potential mediator of depressed leucocyte trafficking.

**Abbreviations**
BDL: bile duct ligation
CCl_4_: carbon tetrachloride
NO: nitric oxide
PG: prostaglandin

**Key points**

- BDL mouse (2 weeks) and rat (4 weeks) models both exhibit clinical and biochemical features that occur in patients with acutely decompensated (AD) cirrhosis whereas the CCl_4_ mouse (10 weeks) model has no phenotypic changes and only an elevated AST on blood testing similar to patients with stable cirrhosis.
- AD patients characteristically demonstrate both significant inflammation and cirrhosis on liver biopsy. CCl_4_ mice and BDL rats are cirrhotic but have no/few inflammatory cells present whereas BDL mice livers have significant inflammation but only mild fibrosis.
- BDL mice and rats share several innate immune defects and clinical characteristics with acutely decompensated cirrhosis patients whereas the chronic CCl_4_ mouse model displayed no clinical or immunological abnormalities.
- We recommend that the combination of clinical, biochemical and immune features observed in BDL rodents make these appropriate models for the study of infection in advanced liver disease. However, only the mice have significantly elevated plasma levels of PGE_2_ as seen in humans.

## Background and aims

Around 0.1% of the European population are estimated to have cirrhosis 1 with an estimated 170 000 deaths per year 2. Irrespective of the underlying cause, decompensation of cirrhosis represents the end stage of the disease process and carries a substantial burden of morbidity and mortality 3. These patients require frequent hospital admission for bacterial infections, which represent a major cause of decompensation and death. These patients are highly susceptible 4 to infection with innate immune dysfunction long considered to represent a major underlying cause of this increased risk 4, 5, 6. In addition, outcome following infection is more severe compared to patients with other chronic conditions 7, 8 and has been shown to be directly related to the development of organ dysfunction 9.

We have recently identified the up regulation of circulating prostaglandin (PG) E_2_ as a potential key mediator underlying immunosuppression in patients with acute decompensation of cirrhosis 10. Previous studies have identified other important factors underlying the poor outcome of infection in these patients. These include reduced leucocyte trafficking to the site of infection, bacterial translocation leading to endotoxin tolerance, elevated circulating nitric oxide (NO) 11, adaptive immune dysfunction, complement activation, malnutrition, propensity to renal failure and cardiovascular collapse 12. A rodent model of liver disease that shared these phenotypic features would greatly assist *in vivo* study of the mechanisms underlying this poor outcome and for testing of potential therapeutics. However, rodent models are unpopular as they poorly reflect the liver disease process in humans which characteristically takes place over 10–30 years 13. Nevertheless, we found previously that both the bile duct ligated (BDL) at 14 days and the carbon tetrachloride (CCl_4_; sampling within 24 h of final injection) mice models both demonstrated an up regulation of circulating PGE_2_ and leucocyte dysfunction similar to that seen in acute decompensation patients. Bile duct‐ligated rats have also been used to study infection and cirrhosis 14.

Our aim was to select a model that was likely to reflect a patient with well‐compensated cirrhosis in which immune function is considered to be broadly intact 15 and use this as our control and reasoned that cirrhotic CCl_4_ mice that had been given 1 week to recover from their last injection might be representative. We had already studied the BDL mouse model at 2 weeks which had shown immune suppression secondary to an up regulation of PGE_2_ 10 and therefore this model was selected to interrogate other potentially important factors in the response to infection in liver disease. As a comparator, we selected the Rat BDL model at 4 weeks as this leads to cirrhosis rather than just mild fibrosis as seen in mice and has been used previously as a model of AoCLF 14. We therefore hypothesized that this would be a more clinically relevant model with the added advantage that, as a larger rodent, it would be able to tolerate sequential plasma sampling pre‐ and post‐infectious/inflammatory insult or therapeutic intervention which is not possible with mice.

We aimed therefore to evaluate three models of rodent liver injury to identify which demonstrated the greatest clinical and immunological phenotypic similar to patients with acutely decompensated cirrhosis. We selected BDL rats at 4 weeks, BDL mice at 14 days and CCl_4_ mice at 10 weeks Our studies previously had used a CCl_4_ model with studies performed the same day as the final CCl_4_ infection to simulate the clinical scenario of acute decompensation of cirrhosis. However, in these studies, we elected to use a model with studies performed 7 days after the final CCl_4_ injection to study a model that we felt was more likely to replicate an outpatient with stable liver disease, referred to as chronic CCl_4_ mice.

## Materials and methods

### Animal maintenance

Rodents were maintained in a 12/12 h light/dark cycle at 22 ± 1°C and given food and tap water *ad libitum* in accordance. Experiments were performed under UK Home Office approval according to the Animals (Scientific Procedures) Act 1986. Furthermore, all animals received human care and that our study protocols complied with University College London's guidelines. Studies were performed in male Sprague–Dawley rats (220–250 g) and C57Bl6/J mice (20–25 g), both from Charles River UK, Margate, UK. Approximately, 135 animals were used in total for these experiments.

### Liver injury

Bile duct ligation/sham procedures were carried out under anaesthesia (isoflurane 1.5%) as described previously. Carbon tetrachloride (CCl_4_; Merck, Darmstadt, Germany) was given subcutaneously (s.c., 1:1 dissolved in olive oil; 1 ml/kg) twice weekly and 300 mg/L phenobarbital added to drinking water. Sham mice received s.c. injections of olive oil. After 14 days for BDL mice, 28 days for BDL rats or 10 weeks for CCl_4_ mice, either peritonitis or paw swelling models were carried out or blood/liver/paws were taken, decalcified (paws only) and prepared for histology or further experimental use. Unlike our previous study, CCl_4_ mice were left for 1 week after the final injection of CCl_4_ prior to experimental study. Antibiotics were not administered to the rodents at any stage.

### Peritonitis and paw swelling models

About 0.1 mg Zymosan A (Sigma‐Aldrich, Homefield Road, Haverhill , Suffolk) in 500 μl was injected i.p. to mice. Peritoneal cavities were washed out 4 h later with leucocytes prepared for flow cytometry and cell‐free inflammatory exudates stored for further analysis. 1% carrageenan was injected intraplantar to rodents (100 μl for rats; 50 μl mice) with equivalent volume saline injected into the contralateral paw. Inflammation was presented as difference in paw thickness over time using gauge (POCO 2T; Kroeplin, GmbH, Surrey, UK) .

### Flow cytometry, cytokines and NO

Flow cytometry analysis was performed using flowjo (Tree Star Inc, Ashland, OR, USA). All samples were analysed on a FACS‐LSRII or Fortessa (both BD Biosciences, Oxford, UK). Leucocytes were incubated with antibodies to F4/80 (clone BM8; eBioscience), murine CD3 (clone KT3; Serotec, Kidlington, UK), CD19 (clone 6C5; Serotec), GR1 (clone RB6‐8C5; BD Pharmingen, San Diego, CA, USA) using respective isotype antibodies and FMOs as controls and compensated for dual labelling. Cytokine expression profiles were measured by dedicated ELISA (TNFα, IL6 and IL10 – mouse eBioscience, San Diego, CA, USA, R&D systems, Abingdon, UK). Samples were run in duplicate. NOx was measured as total nitrite and nitrate in samples deproteinated by ultra‐centrifugation followed by nitrate reductase assay and Griess Reaction and confirmed using chemiluminescence.

### Mouse blood analysis and macrophage isolation/culture

Peritoneal macrophages from healthy animals were isolated as described previously and incubated with/without LPS (*Salmonella Typhosa*, 0.1 μg/ml for 24 h; Sigma‐Aldrich^®^) in the presence of plasma from naïve, sham, CCL_4_ or BDL rodents in cell culture media (complete DMEM; Life Technologies^™^, Paisley, UK) (eBioscience). Samples were run in duplicate.

### Human blood analysis and macrophage isolation/culture

Patient's samples were provided from DASIMAR UKCRN [University College Hospital London Hospitals’ (UCLH) research ethics committee number:08/H0714/8], while healthy volunteers were used as controls. Peripheral venous blood was collected into 5 IU/ml heparin. Plasma was assayed for NOx as described above for mice.

### Extraction and analysis of lipid mediators

Lipid mediators in mice plasma were analysed by liquid chromatography coupled to electrospray ionization tandem mass spectrometry (LC/ESI‐MS/MS) based on protocols published previously 16, 17. Briefly, samples were collected and stored immediately at −80°C. Plasma Samples (250–500 μl) were defrosted on ice and adjusted to 15% (v/v) methanol: water (final volume 4 ml). Internal standards, PGB_2_‐*d*4 (40 ng) and 12‐HETE‐*d*8 (40 ng) (Cayman Chemical Company, Ann Arbor, MI, USA) were added and the pH of resulting solutions adjusted to 3.0 (1m HCL). Acidified samples were immediately applied to preconditioned solid‐phase cartridges (C18‐E; Phenomenex, Macclesfield, UK) and lipid mediators eluted with methyl formate. LC/ESI‐MS/MS analysis was performed on a HPLC pump (Waters Alliance 2695, Hertfordshire, UK) coupled to an electrospray ionization triple quadruple mass spectrometer (Quattro Ultima, Waters, UK). Chromatographic separation was performed on a C18 Luna column (5 μm, 150 × 2.0 mm; Phenomenex) for eicosanoids and a C18 Kinetex column (2.6 μm, 100 × 2.1 mm; Phenomenex) for hydroxy‐ fatty acids. Quantitative analysis was based on multiple reaction monitoring‐based assays as reported 15, 16 with the following additions: 15‐hydroxyeicosatrienoic acid (HETrE) *m/z* 321 > 221, 10‐hydroxydocosahexaenoic acid (HDHA) *m/z* 343 > 153, 14‐HDHA *m/z* 343 > 161, 13‐HDHA *m/z* 343 > 193 and 17‐HDHA *m/z* 343 > 201. Calibration lines were constructed using commercially available standards (Cayman).

### Interventional models: peritonitis and intra‐venous bacterial inoculation

Group B Streptococcus (GBS) (NCTC10/84, serotype V) was grown in Todd Hewitt broth without agitation at 37°C to an OD600 of 0.4, equivalent to 10^8^ colony forming units (CFU)/ml, centrifuged/washed with sterile PBS and injected intraperitoneally (i.p.) at 30 × 10^6^ colony forming units (CFUs) in 300 μl sterile PBS for bacterial killing assays. The nitric oxide synthase (NOS) inhibitors 1400W or L‐NAME (10 mg/kg s.c. or 50 mg/kg po and s.c.; Sigma‐Aldrich^®^) were administered to mice 1 h prior to zymosan or bacterial challenge. Following bacterial challenge, mice were sacrificed at 3 h after GBS injection, heparinized blood taken, centrifuged (10 000*g*, 4°C, 10 min), plated on agar overnight and CFUs counted the following day.

Data from sham and streptococcus was obtained at the same time as experiments previously published (10) as intervention with L‐NAME was performed at the same time to enable us to be adherent to the 3Rs principle of animal research.

### Evaluation of Organ dysfunction

#### Liver/Renal

Blood was collected by intracardiac puncture into heparin and centrifuged (10 000*g*, 4°C, 10 min). Plasma was analysed for liver and renal function tests using the COBAS^®^ INTEGRA 400 plus multianalyser with appropriate diagnostic kits (Roche – Diagnostics, Burgess Hill, UK) or stored at −80°C.

#### Echocardiography

Two‐dimensional images were recorded using echocardiograph (VIVID 7 dimension; GE Vingmed, West Sussex, UK) with epicardial probe (model i13L; GE Vingmed). For mean arterial blood pressure, arterial catheters were inserted under isoflurane (1.5%) and pressure recorded onto a precalibrated PowerLab system (ADInstruments, Oxford, UK) 18.

### Statistical analysis

For calculation of group sizes, from experiments with murine peritonitis, cellular profiles, inflammatory protein expression and lipid mediator production is extremely reproducible. We consider an effect size of ~40% of parameter mean biologically relevant. To enable statistical determination at a *P* < 0.05 in a primary anova screen followed by *post‐hoc* Bonferroni corrected *T*‐test at 90% power, a group size of six animals is necessary with a maximum of five groups per experiment. Applying this approach to humans using human cirrhotic plasma nitrite levels a minimum of *n* = 10/group was required. Statistical analysis was performed using GraphPad Prism 4 (GraphPad Software). For comparisons between multiple groups, one‐way anova with repeated measures was performed followed by Bonferroni post‐test. Comparisons between two groups were made by 2‐tailed (un)paired *t* test. No data were excluded from analysis.

## Results

### Evaluation of organ dysfunction assessment

#### Bile Duct Ligation causes organ dysfunction as opposed to CCl_4_ model of liver injury

BDL mice demonstrated significant weight loss of 20% and mice were hypothermic. Detailed cardiac assessment revealed that peak velocity, stroke volume and heart rate all fell significantly in BDL mice by day 14 (*n* = 12, *P *<* *0.001, Table 1). To exclude hypothermia as a cause of cardiovascular dysfunction, we heated BDLs to the same core temperature as shams. This had no effect on peak velocity or stroke volume but raised heart rate from 431 (15) to 494 (10) (*P* < 0.01) compared to shams rate of 547 (16). Sham operated cardiac values were identical to naive mice (Table 1). Our previous study demonstrated that BDL mice had grossly elevated serum bilirubin values, significant rises in transaminases, reduced serum albumin and creatinine was elevated (listed in Table 1 for comparison). CCl_4_ mice displayed no cardiovascular changes or weight loss and an elevation in AST was the only blood test abnormality. BDL rats showed marked changes in liver blood tests and significant weight loss but remained normothermic.

**Table 1:** Clinical and biochemical data for rodents ± liver injury (*n* = 5–10 animals per group)

	Sham rat	BDL rat	Naïve mouse	Sham mouse	BDL mouse	CCl_4_ mouse
Albumin (g/L)	39 (1.6)	30 (1)**	29 (3)	29 (1)	24 (2)**	30 (1.5)
ALT (IU/L)	60 (13)	91 (11)	20 (9)	26 (2.4)	340 (33)***	42 (4)
AST (IU/L)	87 (23)	443 (60)**	68 (11)	94 (18)	496 (55)	127 (23)*
Total Protein (g/L)	46 (11)	58 (1.3)	41 (3)	43 (2)	32 (2.7)**	42 (6.6)
Glucose (mmol/L)	12 (1)	7.5 (0.3)	15 (0.2)	12 (1)	6 (1)	10 (1.6)
Bilirubin (umol/L)	2 (0.2)	192 (15)***	12 (4)	17 (2.9)	380 (35)***	3 (1)
Creatinine (umol/L)	27 (3)	26 (1.5)	9 (0.7)	11 (0.3)	27 (2.6)**	10 (1)
Urea (mmol/L)	6 (1)	8 (0.6)	9 (0.7)	8 (0.4)	9 (0.9)	8 (1)
Cardiac assessment						
Peak Velocity (m/s)				0.96 (0.04)	0.67 (0.04)***	1 (0.06)
Stroke volume (μl)				49 (2.4)	37 (1.9)***	50 (3.3)
Heart rate (BPM)				547 (16)	431 (15)***	591 (12)
Temperature (°C)	35 (0.8)	36.4 (0.2)	37.8 (0.2)	37.6 (0.1)	35.1 (0.2)**	37.8 (0.1)
Age at surgery	–	–	11 weeks	11 weeks	11 weeks	11 weeks
Weight at experiment (g)	455 (6)	375 (10)	27.8 (0.6)	27 (0.4)	19.4 (0.3)***	31 (0.4)
Plasma Nitrite (μm)	9.2 (1)	51 (16)		50.5 (9.6)	91 (12)**	22 (4)

*P <* *0.05; **P <* *0.01; ***P <* *0.001, *t*‐test for BDL or CCl_4_ vs naïve or sham mice.

#### Liver histology

**Figure 1:**
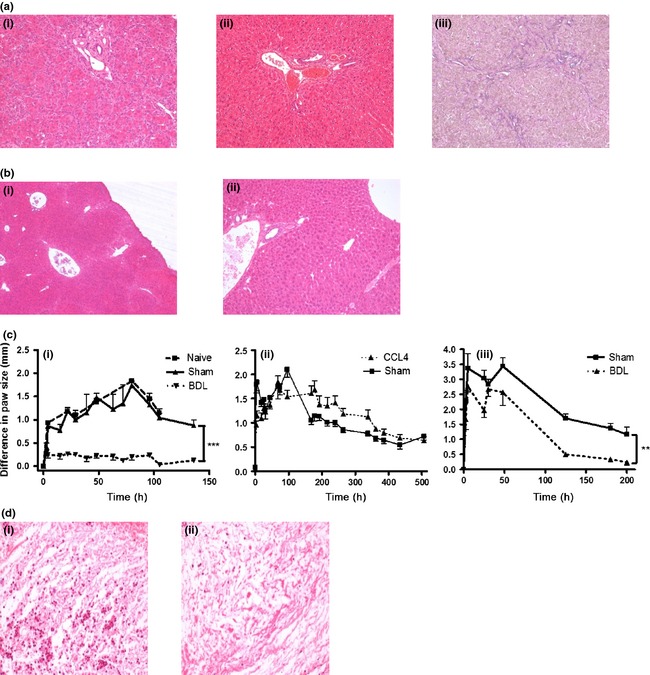
(a‐i) Haematoxylin and Eosin (HE)‐stained histological sections of the portal tract of liver from bile duct ligated (BDL) rats showing ductular reaction and scant inflammation. (a‐ii) HE‐stained histological sections from Sham rats (28 days post procedure). (a‐iii) Elastica Van Gieson (EVG) staining of BDL rat liver demonstrating bridging fibrosis. (b‐i) HE‐stained histological sections of the portal tract of liver from CCl_4_ mice (at 10 weeks, samples taken one week after final CCl_4_ injection) showing nodular parenchyma with no inflammation and (b‐ii) sham mice. (c) Effect of carrageenan‐induced paw oedema (1%, 50 μl intra plantar injection in mice, 100 μl in rats) in (i) BDL mice (14 days post procedure, *n* = 9), (ii) CCl_4_ mice (*n* = 6) and (iii) BDL in rats (*n* = 6). Graphs show difference in paw size between carrageenan‐injected and saline‐injected paw. (d) Representative sections through rat paw 4 h following carrageenan from (i) sham and (ii) BDL animals. All histology slides at magnification ×20. For carrageenan experiments 3–6 animals per group were used. Data are represented as mean ± SEM. *** *<* *0.01 ***P <* *0.001, anova.

We have previously demonstrated that histological analysis of mouse BDL liver revealed a chronic inflammatory infiltrate of lymphocytes, eosinophils and plasma cells but no fibrosis 10. Heamatoxylin and eosin (H&E) (Fig. 1a‐i) and elastic van gieson (EVG) (Fig. 1a‐iii) staining of BDL rat livers demonstrated cirrhosis, marked ductular reaction but little inflammation was observed whilst cirrhotic nodules but no without inflammation were seen in CCL_4_ mouse livers (Fig. 1b‐i).

### Evaluation of inflammatory response

#### Both BDL mice and rats exhibit depressed acute inflammatory response to carrageenan‐in‐paw swelling

Both BDL rodent models demonstrated a significantly reduced acute inflammatory response to carrageenan paw swelling (especially BDL mice) whereas CCl_4_ mice responded similar to shams (Fig. 1c i–iii). Histological section of the paws confirmed a marked reduction in inflammatory cell infiltrate in the BDL rodents (Fig. 1d i and ii).

#### BDL rats but not Chronic CCl_4_ mice have elevated plasma eicosanoid levels

Lipidomic analysis of plasma from BDL rats revealed a modest non‐significant elevation in PGD_2_ and 15‐HETE and significant rises in 13‐HODE, 15R‐HETE 14‐HDHA and 17‐HDHDA. However, there were no differences in plasma eicosanoid levels between sham and chronic CCl_4_ mice (Table 2). Although we screened for 47 metabolites products of COX and LOX mediated pathways, only 17 mediators were detected as shown in Table 2.

**Table 2:** LC/ESI‐MS/MS analysis of plasma from sham & CC_L4_ mice and sham and BDL rats for the 47 known metabolites of COX and LOX enzymes (ng/ml). (*n* = 5–6 mice per group)

Measured metabolite	Sham mice	CCL4 mice	Sham rat	BDL rat
PGE_2_	0.73 (0.09)	0.9 (0.24)	0.34 (0.04)	0.52 (0.15)
PGD_2_	0.31 (0.14)	0.18 (0.1)	0.06 (0.01)	0.12 (0.04)
PGF_2α_	0.88 (0.39)	0.67 (0.39)	0.34 (0.15)	0.44 (0.2)
TXB2	2.24 (1.12)	1.67 (0.96)	0.91 (0.41)	0.83 (0.42)
9‐HODE	9.2 (5.3)	11 (4.9)	3.8 (1.7)	5.5 (2.5)
13‐HODE	29.3 (17)	34.8 (16)	10.6 (4.7)	66.1 (30)
12‐HEPE	20.5 (12)	14.1 (6.3)	0.9 (0.4)	0.7 (0.3)
5‐HETE	2.8 (1.6)	4.2 (1.9)	1.1 (0.5)	0.9 (0.4)
8‐HETE	2.9 (1.7)	4.6 (2.1)	1 (0.4)	0.7 (0.3)
11‐HETE	2.5 (1.4)	3 (1.4)	1.5 (0.6)	0.6 (0.3)
15‐HETE	4.2 (2.4)	3 (1.7)	1.9 (0.8)	3.5 (1.6)
12‐HETE	276 (159)	378 (170)	37.3 (17)	25.2 (11)
15‐HETrE	0.5 (0.3)	0.8 (0.4)	0.2 (0.1)	1.3 (0.6)
10‐HDHA	1.2 (0.7)	1.3 (0.6)	0.04 (0.0)	0.3 (0.2)
14‐HDHA	12 (7)	12.4 (5.5)	0.5 (0.2)	3 (1.3)
13‐HDHA	0.5 (0.3)	0.8 (0.4)	0.2 (0.1)	0.6 (0.3)
17‐HDHA	2.8 (1.6)	4.5 (2)		14 (6.3)

PG, Prostaglandin; TX, Thromboxane; HODE, Hydroxyoctadecaenoic acid; HETE, Hydroxyeicosaenoic acid; HETEr, hydroxyeicosatrienoic acid; HDHA, Hydroxydocosahexaenoic acid; HEPE, hydroxy eicosapentaenoic acid.

#### Plasma from BDL rats exhibits a partial PGE_2_‐mediated immune suppressive effect on macrophage function whereas plasma from chronic CCl_4_ mice has no immune suppressive effect

**Figure 2:**
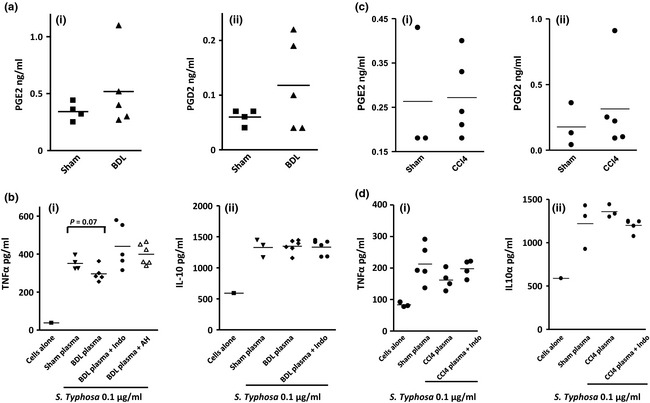
(a) Lipidomic analysis demonstrated that (i) PGE_2_ and (ii) D_2_ were minimally elevated in BDL rats compared to shams. (b) Plasma from BDL rats induced a partial reduction in (i) LPS‐induced TNFα production from naïve mice peritoneal macrophages compared to plasma from shams which was attenuated when the EP1–3/DP selective inhibitor AH6809 was added *in vitro* or if plasma from BDL rats treated with indomethacin (Indo) was added. (b) Plasma from BDL rats had no effect on (ii) IL10 secretion from naïve mice peritoneal macrophages. (c) (i) PGE_2_ and (ii) iD_2_ levels were minimally elevated in plasma from Chronic CCl_4_ mice but (d) this plasma had no effect on peritoneal macrophage LPS‐induced (i) TNFα or (ii) IL10 production function compared to sham plasma.

Lipidomic analysis revealed that PGE_2_ and D_2_ was minimally elevated in BDL rats compared to shams (Table 2 & Fig. 2‐ a i and ii). In addition, their serum albumin concentration, which we have demonstrated modulates the immunosuppressive effects of PGE_2_ 10, was substantially reduced [from 39 (1.6) to 30 (1) g/L, mean (SEM) see Table 1]. When plasma from BDL rats was added to mouse peritoneal macrophages we observed a partial reduction in LPS‐induced TNFα production compared to plasma from shams. This partial effect was attenuated when the EP1‐3/DP selective inhibitor AH6809 was added *in vitro* or if plasma from BDL rats treated with the non‐selective cyclo‐oxygenase inhibitor indomethacin was added (Fig. 2b‐i). There was no effect of BDL rat plasma on macrophage IL10 production (Fig. 2b‐ii).

Chronic CCl_4_ mice also exhibited a modest but not significant rise in PGE_2_ but not D_2_ (Table 2, Fig. 2c‐i and ii) and the serum albumin was unchanged compared to shams (Table 1). Plasma from these animals had no anti‐inflammatory effect on mice peritoneal macrophages (Fig. 2d‐i and ii).

We have previously shown that the BDL mouse model demonstrated PGE_2_ mediated immune suppression 10. In view of our overall work, we decided to use the mouse BDL model as our model of acute decompensation of liver disease and use the chronic CCl_4_ mice as a model of stable cirrhosis.

### Comparison of response to peritonitis in experimental models of acute decompensation and stable cirrhosis

#### Elevated nitric oxide (NO) via inducible nitric oxide synthase (iNOS) mediates defective leucocyte trafficking and function in bile duct‐ligated rodent models of Liver injury

**Figure 3:**
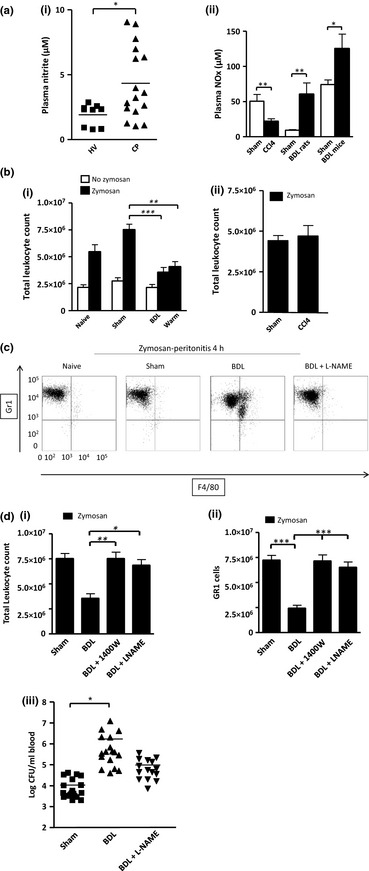
(a‐i) Plasma nitrite levels in patients admitted to hospital with complications of cirrhosis (CP) compared to healthy volunteers (HV). (a‐ii) Plasma NOx levels in sham and liver injury rodents (*n* = 6 for each group). (b) Total leucocyte count following zymosan‐induced peritonitis in naïve, sham & BDL mice ± zymosan (Z; 0.1 mg at 4 h) and BDL mice warmed (warm) to the same core temperature as shams (b‐i) as well as CCl_4_ mice (b‐ii); *n* = 8–10 – data taken from 3 consecutive experiments. (c) Representative flow cytometry traces of GR1 and F4/80 labelled peritoneal macrophages 4 h after zymosan in naïve, sham, BDL and BDL + LNAME mice. These cells are gated on CD3 negative CD19 negative and CD 11B positive to exclude T and B cells. (d‐i and ii) Zymosan (0.1 mg) was injected, i.p. to naïve, sham and BDL mice with/without NOS inhibition [1400W (iNOS inhibitor), L‐NAME], with peritoneal PMNs showing the greatest reduction in cell numbers with full reversal seen in the presence of NOS inhibition. (d‐iii) Colony forming units detected in sham or BDL mouse blood at 3 h following i.p. injection of Group B streptococcus (GBS, 30 million units per mouse). BDL mice were treated with or L‐NAME (50 mg/kg) 30 mins before bacteria injection. Sham and BDL data as previously shown^10^, reproduced for this figure as experiments performed using L‐NAME were performed together to minimize animal usage. *P <* *0.05 **P <* *0.01 ***P <* *0.001, anova or *t*‐test where appropriate.

Plasma NOx was elevated in patients admitted with acute or chronic liver failure (AoCLF) compared to healthy volunteers and in bile duct‐ligated rodents but not in CCl_4_ mice compared with sham animals (Table 1, Fig. 3a‐i & ii).

We found that following intraperitoneal zymosan BDL mice demonstrated reduced cell trafficking compared to shams (*P* < 0.01) (Fig. 3b‐i). As BDL mice were hypothermic, we warmed them to 37.5°C prior to zymosan but this had no significant effect on leucocyte trafficking (Fig. 3b‐i). Conversely, cell trafficking following zymosan was similar between CCl_4_ mice and their respective shams (Fig. 3‐ii). Flow cytometric analysis confirmed that GR1 cell numbers were principally affected in the BDL mice (Fig. 3c). This reduced leucocyte trafficking was completely reversed following NO synthase (NOS) inhibition by both the non‐selective inhibitor NOS inhibitor L‐NAME and the highly selective iNOS inhibitor (1400W) (Fig. 3d‐i and ii).

Finally BDL mice were injected with live Group B streptococcus (GBS, NCTC10/84, serotype V). L‐NAME partially restored bacterial killing towards sham levels (Fig 3d‐iii).

## Discussion

Infection is one of the major causes of decompensation and death in advanced liver disease and therefore the development of more effective treatments against this represents a major challenge for hepatologists 12. Several defects in the innate immune response have been demonstrated in these patients including our recent identification of PGE_2_ mediated leucocyte dysfunction 3. *In vivo* testing of novel treatments is extremely difficult in patients with advanced liver disease and therefore development of representative rodent models of the associated innate immune dysfunction is extremely important. As well as immune dysfunction, extra hepatic organ dysfunction has emerged as a key indicator of poor prognosis following infection 9 and therefore an ideal model would exhibit both of these features. In this study, we have shown that BDL mice and rats share several innate immune defects and clinical characteristics with acutely decompensated cirrhosis patients whereas the chronic CCl_4_ mouse model displayed no clinical or immunological abnormalities (see Table S1). Although BDL mice have little histological similarity to acutely decompensated cirrhosis unlike the rats which demonstrate cirrhosis, they do have significantly elevated plasma levels of PGE_2_ as seen in humans.

The BDL mice have severe liver dysfunction, renal impairment and low blood glucose levels. They demonstrate sarcopenia and cardiovascular dysfunction. These are characteristic clinical features of acutely decompensated cirrhosis. It should be noted that, in keeping with previously published data 19, cardiac output is actually reduced whereas most commonly a high cardiac output 20 is observed in acute decompensation patients. Although increasingly a low output state has been recognized and correlates with a poor prognosis.

Bile duct ligation in rats induced cirrhosis and severely deranged liver enzymes, NO levels were significantly elevated and carrageenan‐in‐paw responses were significantly dampened. However, PGE_2_ levels were only minimally elevated and we observed that plasma from BDL rats could only induce a small reduction in macrophage function. We attribute this to the slight increase in plasma PGE_2_ combined with the reduction in albumin which modulates the effects of PGE_2_ as albumin is known to both bind and catalyse E‐series prostaglandins 21. Other groups have successfully used endotoxin treated BDL rats as model of acute or chronic liver failure. Although PGE_2_ is highly likely to be elevated under such circumstances these models are short term (3 h) 14 which would not allow time for detailed immune assessment.

Chronic CCl_4_ mice had slightly elevated PGE_2_, normal plasma NO and albumin concentration and normal cardiac and renal function, similar to stable cirrhosis patients (Child Pugh A or low MELD score). Therefore, whilst appropriate for studying mechanisms that underlie fibrosis this is not a relevant model for the study of immune dysfunction. It is possible that we may have observed a different result had we used BALB/c mice which develop fibrosis secondary to CCl_4_ more readily than C57BL/6 inbred mice 22. However, for our original BDL model studies we had used C57BL/6 mice and therefore we continued with this breed. This is in marked contrast to our previous study in which PGE_2_ levels were elevated acutely following CCl_4_ injection 10. This subtle difference in methodology should be considered when using this model. If the model is extended to up to 15 weeks decompensation with ascites occurs and immune responses are highly likely to be altered but again we felt that the associated high mortality (70%) with this model was unacceptable 23.

We demonstrate that elevated nitric oxide (NO) impaired cell trafficking in BDL mice but that inhibition only partially reversed the impaired bacterial killing observed in these mice compared to shams. Therefore, it is not clear whether this improvement in trafficking confers any functional benefit. NO has been shown to be elevated in patients with advanced cirrhosis and this correlated with disease severity 24. *In vivo* neutrophil migration was decreased in cirrhotic patients with previous episodes of bacterial infection compared with non‐infected patients using a skin window technique suggesting that deficient neutrophil recruitment to the infection site may contribute to increased bacterial infections in cirrhotic patients with advanced liver disease 25. Further investigation is required to establish whether NO mediates this process in acute decompensation patients and furthermore whether inhibition has a demonstrable effect on the impaired response to infection in these patients.

The two major criticisms of the 2 week BDL mice model are the absence of significant liver fibrosis and that it is a defined surgical procedure rather than the chronic or repetitive inflammatory insults that occur in alcohol, viral hepatitis and Non‐Alcoholic Steatohepatitis (NASH), which account for approximately 90% of liver disease within the UK 26. Mouse models of NASH 27 and alcohol 13 induced liver damage do not reliably cause cirrhosis or advanced liver dysfunction and were therefore not tested during this study. We have shown that the chronic inflammatory injury combined with the liver dysfunction observed in this model create a similar immune suppressive phenotype to that observed in acute decompensation patients. It would therefore appear that liver dysfunction rather than fibrosis *per se* is necessary for development of this phenotype. It has been shown that BDL mice at 6 weeks develop cirrhosis 28 but we found that these mice lost significant amounts of weight leading to an unacceptable mortality. Equally we have focused on only two mediators of immune dysfunction and there are clearly others; further work is required to determine whether BDL mice can be used to study other putative mediators or mechanisms, although rapid onset of intestinal bacterial overgrowth (within 1 day) has been demonstrated in these mice 29 as opposed to 10 weeks for CCl_4_ mice.

We conclude that BDL mice and rats are not simply models of cholestatic liver injury but may be used to study mechanisms underlying poor outcome from infection in AD although rats demonstrated only minimally elevated plasma PGE_2_. We have also identified elevated NO as a potential mediator of depressed leucocyte trafficking in these models although reversal of this process did not restore impaired bacterial killing in mice.

## Supporting information

Additional Supporting Information may be found at onlinelibrary.wiley.com/doi/10.1111/liv.12876/suppinfo
