# Supplementary material for: Bile duct‐ligated mice exhibit multiple phenotypic similarities to acute decompensation patients despite histological differences
Source: Liver Int. 2015 Jun 22;36(6):837–46. doi: 10.1111/liv.12876 (PMC4869675; doi:10.1111/liv.12876)
Supplement: Supplementary file 1 [file LIV-36-837-s001.doc]

**Supplementary Table 1.** A comparison of the 3 mouse models with respect to the phenotypic characteristics considered to underlie poor response to infection observed in Acute Decompensation patients

|  | **Acute decompensation**  **Patients** | **BDL MICE (2weeks)** | **BDL RATS**  **(4 weeks)** | **CCL4 MICE**  **(10 weeks)** |
| --- | --- | --- | --- | --- |
| **Histological features** | | | | |
| **Fibrosis** | +++ | + | +++ | +++ |
| **Inflammation** | +++ | +++ | + | - |
| **Clinical features predisposing to poor outcome following infection** | | | | |
| **Severe Liver dysfunction** | +++ | +++ | +++ | - |
| **Renal Impairment** | ++ | + | - | - |
| **Cardiovascular dysfunction** | +++ | +++ | ? | - |
| **Sarcopenia** | +++ | ++ | + | - |
| **Features relevant to immune dysfunction** | | | | |
| **Elevated circulating PGE_2_** | +++ | +++ | + | - |
| **Defective neutrophil trafficking** | ++ | ++ | ? | - |
| **Elevated circulating Nitric Oxide** | ++ | ++ | ++ | - |
| **Impaired Bacterial killing** | ++ | ++ | ? | ? |
